# Porous silicon based intravitreal platform for dual-drug loading and controlled release towards synergistic therapy

**DOI:** 10.1080/10717544.2018.1486474

**Published:** 2018-07-11

**Authors:** David Warther, Ying Xiao, Fangting Li, Yuqin Wang, Kristyn Huffman, William R. Freeman, Michael Sailor, Lingyun Cheng

**Affiliations:** a Department of Chemistry and Biochemistry, University of California, San Diego, CA, USA;; b Department of Ophthalmology, Jacobs Retina Center at Shiley Eye Institute, University of California, San Diego, CA, USA;; c Provincial Hospital Affiliated to Shandong University, Jinan City, Shandong Province, China

**Keywords:** Dual-drug loading, intravitreal drug delivery, porous silicon, dexamethasone, daunorubicin, controlled release

## Abstract

The number of blind and low vision persons in the US is projected to increase to 5.68 million by 2020. The eye diseases causing loss of vision are life-long, chronic, and often need protracted presence of therapeutics at the disease site to keep the disease in remission. In addition, multiple pathologies participate in the disease process and a single therapy seems insufficient to bring the disease under control and prevent vision loss. This study demonstrates the use of porous silicon (pSi) particles sequentially loaded with daunorubicin (DNR) and dexamethasone (DEX) to create a synergistic intravitreally injectable dual-drug delivery system. DEX targets chronic inflammation while DNR inhibits excessive cell proliferation as well as suppresses hypoxia-inducible factor 1 to reduce scarring. This pSi-based delivery system releases therapeutic concentrations of DNR for 100 days and DEX for over 165 days after a single dose. This intravitreal dual-drug delivery system is also well tolerated after injection into the rabbit eye model, attested by ocular biomicroscopy, ocular tonometry, electroretinography, and histology. This novel dual-drug delivery system opens an attractive modality for combination therapy to manage refractory chorioretinal diseases and further preclinical studies are warranted to evaluate its efficacy.

## Introduction

Chorioretinal diseases are major blinding diseases, which need protracted treatment to suppress relapses and combination therapy to target their multiple pathologies, such as the overexpression of vascular endothelial growth factor (VEGF) and persistent low-grade inflammation as seen in age-related macular degeneration (AMD) and diabetic retinopathy (Aiello et al., 1994; Funatsu et al., 2002). Nowadays, intravitreal injection of anti-VEGF agents has become the standard treatment for wet AMD and diabetic macular edema (DME) (Rofagha et al., 2013; Wells et al., 2016). However, not all patients respond favorably to these interventions that solely neutralize VEGF (Krebs et al., 2013). In addition, a decade of history of repeated intravitreal injections of anti-VEGF antibodies has demonstrated that disciform scarring and inner retina atrophy at the macula resulting in poor vision is an inevitable outcome (Batman & Ozdamar, 2010; Hwang et al., 2011; Daniel et al., 2014; Young et al., 2014) in a large number of patients. We believe that simultaneous deployment of both anti-scarring/VEGF-downregulating and anti-inflammatory drugs will yield a more favorable treatment response. Persistent low-grade inflammation plays an important role in many retinopathies including AMD and various stages of diabetic retinopathy (Telander, 2011; Antonetti et al., 2012). Inflammatory chemokines not only create a neurotoxic milieu, but also recruit macrophages and lymphocytes to the vitreoretinal interface or subretinal space to participate in the process of fibrovascular proliferation (Grossniklaus et al., 2002; Semkova et al., 2011). In addition, prolonged suppression of intraocular VEGF levels by anti-VEGF drugs may trigger overexpression of connective tissue growth factor that subsequently breaks the angio-fibrotic balance and promotes fibrosis (Kuiper et al., 2008; Van Geest et al., 2012).

Anti-inflammatory agents, such as steroids, have been used for the management of AMD and DME even though the ocular side effects are of high concern for both physicians and patients (Maturi et al., 2016). Dexamethasone (DEX) is a steroid well-known for its potent anti-inflammatory effect (Wang et al., 2011). The currently marketed DEX implant, Ozurdex, has demonstrated beneficial therapeutic effect on various macular edema (Aroney et al., 2016). However, a recent clinical trial revealed that Ozurdex did not prevent the reoccurrence of proliferative retinopathy (PVR) (Banerjee et al., 2017). This again highlights the pharmacodynamic complexity of each retinal disease and its management as well as the unmet need for combination therapy. Daunorubicin (DNR) is an anthracycline antibiotic that stops cell proliferation through a few different proposed mechanisms, such as intercalating DNA, inhibiting topoisomerase II, and interacting with the DNA-topoisomerase II complex (Gewirtz, 1999). A single intraocular dose of DNR has demonstrated an anti-proliferative effect against PVR in both experimental and clinical studies (Wiedemann et al., 1998; Hui & Hu, 1999). More importantly, DNR adversely affects multiple facets of the synthesis of HIF-1 (hypoxia-inducible-factor 1) that is upstream of VEGF production (Yamazaki et al., 2006; Hede, 2009). Therefore, inhibiting HIF-1 with DNR can block VEGF production, which is an alternative strategy to be explored for downregulating VEGF. We have previously demonstrated that the short vitreous half-life and narrow therapeutic window of DNR (Wiedemann et al., 1983; Santana et al., 1984; Kwak & D'Amico, 1992) can be largely rectified by incorporating the drug into a slow-release delivery system (Hou et al., 2015). Over the past few years, delivery systems with the ability to simultaneously release multiple drugs have been of growing interest, particularly to circumvent multi-drug resistance (Hu & Zhang, 2012) and to simultaneously deliver hydrophobic and hydrophilic drugs (Su et al., 2014). So far, these systems have merely presented the concept but lack the ability for the long-term delivery of multiple drugs. Most of these systems provide only a few hours to a few days of relevant drug levels. There is a strong need for a practical, long-term, dual-drug delivery technology.

In recent years, porous silicon (pSi) has been investigated as a drug delivery platform (Anglin et al., 2008; Kumeria et al., 2017) and for the sensing of drug release (Xu et al., 2017a) due to its tunable pores for various sized payloads and its photonic properties in different biological conditions. We have demonstrated that hydrosylilated or oxidized pSi is well tolerated after intravitreal injection and can provide sustained delivery of either DNR or DEX (Cheng et al., 2008; Chhablani et al., 2013; Hartmann et al., 2013; Hou et al., 2016). The pSi particles in the vitreous degrade into silicic acid that is cleared from the eye along with the ocular fluid turnover (Nieto et al., 2013). We hypothesized that properly functionalized pSi could be dually loaded with the model drugs, DNR and DEX, to create a platform to simultaneously deliver two drugs in a controlled release manner for synergistic effect.

## Materials and methods

### Synthesis of pSi microparticles

pSi microparticles were prepared by electrochemical etch of highly doped, (100)-oriented, P^++^-type silicon wafers (boron-doped, 1.04 × 10^−3^ Ω.cm resistivity) purchased from Virginia Semiconductors or Siltronix. The wafers were mounted into a 54 cm^2^ etch cell fitted with a platinum counter electrode. New wafers were cleaned as follows prior to any actual porous layer etch. The first porous layer was etched in 150 ml of a 3:1(vol/vol) solution of 48% aqueous hydrofluoric acid (HF)/absolute ethanol (EtOH) under a current of 100 mA cm^−2^ for 1 min. The porous layer was then dissolved by a 2 N KOH solution in water. The cleaned wafer was finally rinsed with deionized water 3 times, ethanol 3 times, and carefully dried under nitrogen. The porous material was then created through an electrochemical etch in 240 ml of a 1:1 (vol/vol) solution of 48% HF/EtOH at a continuous current of 30 mA cm^−2^ for 960 s followed by a pulse of current at 176 mA cm^−2^ for 0.3 s. After allowing the electrolyte to homogenize by stopping the current for 1 s, 30 mA cm^−2^ were applied for an additional 960-s period. The resulting porous layer was then washed once with EtOH and lifted off by electropolishing in a 1:29 solution of 48% HF/EtOH for 400 s at a current of 6 mA cm^−2^. The porous material obtained through each cycle was stored in EtOH in individual glass vials (30 ml). Etching and electropolishing procedures were repeated up to 8 times per wafer. The pSi particles were obtained by ultrasound fracturation of the pSi films (20 µm thickness) in EtOH in an ultrasonic cleaner. The films were sonicated for 15 min and the material was allowed to settle down. The supernatant was removed and the material was washed twice with EtOH then sonicated for an additional 15-min period. After sedimentation and removal of the supernatant, the particles were washed with EtOH until the supernatant remained clear (absence of very small particles).

### pSi oxidation

The harvested pSi particles were thermally oxidized in air immediately after sonication inside a muffle furnace (Thermo Fisher Scientific, Pittsburg, PA) in order to form porous silicon dioxide (pSiO_2_) microparticles. The particles were heated from room temperature to 800 °C at a rate of 10 °C per minute then kept at 800 °C for 1 h. The furnace was then allowed to freely cool to room temperature. After oxidation, the pSiO_2_ particles were stored dry in glass vials.

### Characterization of pSi particles

pSiO_2_ particles were suspended in EtOH. One drop of the suspension was poured on a glass microscope slide and the solvent was allowed to evaporate. The particles were imaged on a bright field microscope mounted with an ×5 lens. Four randomly chosen independent fields of view (a total of at least 200 particles) were imaged for each sample and the images were then processed using ImageJ (Schneider et al., 2012). Each individual particle was manually delimited and the size of each zone was calculated against a reference given by a standard stage micrometer (100 × 0.01 = 1 mm) imaged under the same conditions. All data from each sample were then imported to Excel and a statistical evaluation of the average size and standard deviation were automatically calculated. Scanning electron microscope (SEM) images were obtained by using Phillips XL30 field emission electron microscope to reveal the pore structure on the surface of the particle. Nitrogen adsorption/desorption experiments were run on a Micromeritics Asap 2020. The specific surface area, pore size diameter, and total pore volume parameters were calculated from nitrogen adsorption/desorption isotherms of particles with the application of the Brunauer-Emmett-Teller (BET) and Barrett-Joyner-Halenda (BJH) methods.

### Functionalization of pSiO_2_ particles

The resulting pSiO_2_ particles were suspended into commercial Tris buffer (pH 7.4, 1×) at a concentration of 10 mg/ml for surface activation. After stirring for 2 h, the particles were recovered by centrifugation at 8000 rpm for 2 min. The buffer was removed and the particles were rinsed 3 times with deionized water, 3 times with EtOH, and dried under vacuum. The activated particles were suspended into EtOH (120 μl/mg of particles) and stirred with 3-aminopropyltrimethoxysilane (Sigma-Aldrich Corp., St. Louis, MO, USA; 2.5 μl/mg of particles) at room temperature. After stirring overnight, the particles were centrifuged at 8000 rpm for 2 min and washed 3 times with EtOH, and dried under vacuum resulting in alkylamine-modified particles.

**Figure 1.**
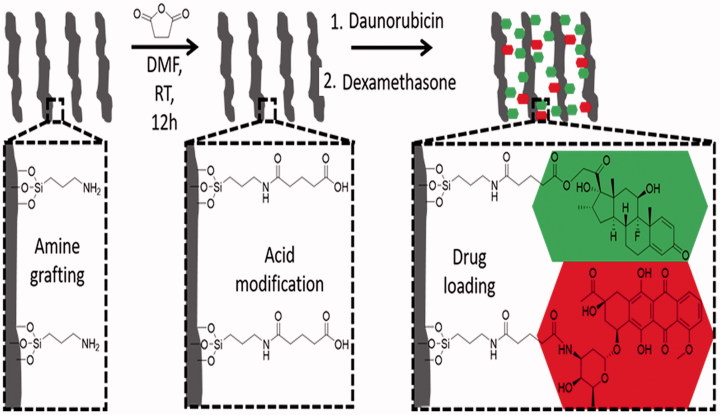
Sketch of drug loading for DEX and DNR. The first step demonstrates a NH_2_ functionalized pSi surface (left sketch) and the second step is showing a COOH terminated pSi surface (middle sketch). The third step demonstrates the conjugation of DEX and DNR to the pSi surface via ester (carbon–oxygen) bond or amide (carbon–nitrogen bond) (right sketch).

Then, 330 mg of amine-grafted pSiO_2_ particles (pSiO_2_–NH_2_) were reacted with 1.65 g of succinic anhydride (99%, Sigma-Aldrich Corp.) in 33 ml of anhydrous N,N-dimethylformamide (Sigma-Aldrich Corp.) at room temperature overnight, washed 3 times with EtOH, and dried under vacuum in order to obtain a succinic acid functionalized surface (pSiO_2_–NH–COOH) as shown in Figure 1.

### Drug loading to pSiO_2_ microparticles

DNR and DEX were covalently loaded into pSiO_2_ particles successively. First, 15.6 ml of a 136 mM solution of N-(3-dimethylaminopropyl)-N′-ethylcarbodiimide hydrochloride (EDC, Sigma-Aldrich) in phosphate buffered saline (PBS)/DMSO (9/1, vol/vol) was mixed with 15.6 ml of a 13 mM solution of N-hydroxysulfosuccinimide (sulfo-NHS) in PBS/DMSO (9/1, vol/vol); 200 mg of pSiO_2_–NH–COOH particles were added to the solution and was stirred for 20 min. Then, DNR was coupled to the activated surface (Figure 1, step 3) by the addition of 960 μl aqueous solution of 10 mg/ml DNR hydrochloride (Tocris Biosciences, Bristol, UK) to the particle mixture. The mixture was stirred for 2 h at room temperature in the dark to form pSiO_2_-DNR microparticles. After the DNR loading procedure, the particles were pelletized by centrifugation at 8000 rpm for 2 min and carefully washed twice with DI water and four times with EtOH until the washing solution was transparent in order to remove unloaded drug and any excess cross linkers. Finally, the resulting particles were dried under vacuum.

Subsequently, 121 mg of pSiO_2_-DNR were suspended in 15 ml of CH_2_Cl_2_. 180 mg of dicyclohexylcarbodiimide (DCC) and 40 mg of 4-N,N-dimethy-lamminopyridine (DMAP) were added to the suspension and the mixture was rotated at room temperature for 20 min in the dark; 70 mg of DEX was added to the mixture system and stirred at room temperature for 7 days in the dark. As CH_2_Cl_2_ freely evaporates over time, 2 ml was added at day 4 to prevent the reaction mixture from getting dry. After reaction, 5 ml of EtOH was added to help dissolve the remaining reactants. The particles were recovered by centrifugation at 8000 rpm for 2 min, carefully washed six times with EtOH to remove unloaded drug and reaction side products, and finally dried under vacuum. At this step, DEX was coupled to the remaining carboxylic group on surface of pSi (Figure 1, step 3). All resultant dual-drug loaded samples were stored at –20 °C to avoid degradation.

For comparison, drug loading was also conducted by loading DEX first and then DNR. 77 mg of pSiO_2_–NH–COOH were suspended in 12 ml of CH_2_Cl_2_; 139 mg of DCC and 31 mg of DMAP were added to the suspension and the mixture was stirred for 20 min in the dark; 54 mg of DEX was then added and the mixture was stirred at room temperature for 7 days in the dark; 2 ml CH_2_Cl_2_ was added at day 4 to prevent the reaction mixture from getting dry. After the reaction, 5 ml of EtOH was added to help dissolve the remaining reactants. The particles were recovered by centrifugation (8000 rpm, 2 min), washed six times with EtOH, and dried under vacuum. Subsequently, 3.52 ml of a 136 mM solution of EDC in a mixture of PBS/DMSO (9/1, vol/vol) was mixed with 3.52 ml of a 13 mM solution of sulfo-NHS in PBS/DMSO (9/1, vol/vol); 45.1 mg of DEX loaded particles were added to the solution and the mixture was stirred for 20 min. Then, 216.5 μl of a 10 mg/ml solution of DNR in DI water was added to the particles and the mixture was stirred for 2 h at room temperature in the dark. After reaction, the particles were recovered by centrifugation (8000 rpm, 2 min), washed six times with EtOH, and finally dried under vacuum.

### Fourier-transform infrared (FTIR) spectroscopy and thermogravimetric analysis (TGA)

The successful surface grafting and covalent drug attachments were determined by FTIR spectroscopy in an attenuated total reflectance mode in a Nicolet 6700 with Smart-iTR spectrometer (Thermo-Scientific); drug loading efficiency was further confirmed by TGA. The samples (∼5 mg) were placed in a 90 μl alumina sample cup. Samples were heated at a constant rate of 10 °C/min from 30 to 800 °C under air atmosphere with a purge rate of 10 ml/min using a Q600 simultaneous TGA/DSC apparatus (TA Instruments, Newcastle, DL). Before heating, the initial weight of each sample was measured six times over 1 min and the average value was used to normalize the remaining weight of the sample during the heating process. After reaching 800 °C, the samples were allowed to cool to 30 °C at a rate of 30 °C/min. The total loss of weight was calculated by the difference of the weight at 130 °C during the heating and cooling phases.

### *In-vitro* release in sink condition

DNR and DEX release from the microparticles *in vitro* was evaluated in PBS. Briefly, 6 mg of DNR/DEX-loaded pSiO_2_ particles were weighed into a 4 ml clean glass vial. A volume of 1.5 ml PBS was added and the tube was vortexed to homogenize the particle suspension. The samples were capped, placed in an incubator at 37 °C, and continuously agitated with a mini laboratory roller. Every 24 h, the vial was centrifuged and 1.3 ml of the supernatant was sampled and replaced with an equal volume of fresh release medium. Based on the drug loading data and our previous studies, roughly 300 µg of DNR was loaded into 6 mg of the pSiO_2_ particles. DNR solubility in water is 627 µg/ml (https://www.drugbank.ca/drugs/DB00694); therefore, DNR will never reach its saturation point. The calculated Dex loaded into 6 mg of the pSiO_2_ particles was roughly 120 µg. Dex has a much lower solubility in water (50 µg/ml, https://www.drugbank.ca/drugs/DB01234) than DNR and the pSiO_2_ rendered its release much slower than its natural dissolution rate (Wang et al., 2014); 1.5 ml of dissolution medium is an adequate volume based on our previous studies, otherwise, drug levels in the release medium will be too low to reliably detect. The supernatant samples were stored at −80 °C until analysis.

### Preparation of the samples for MS/MS analysis

Samples from day 1, day 2, day 4, day 7, day 10, then every 5 days to day 165, and then every 10 days until day 305 were analyzed by HPLC/MS/MS. In brief, 600 μl of release medium was mixed with 500 μl of EtOH in 1.5 ml Eppendorf tubes to dissolve all the drug that could have precipitated during the freeze/thaw process. The mixture was centrifuged (15,000 rpm, 10 min) to remove any insoluble material from the samples. 1.1 ml of the supernatant was sampled into a 2 ml glass vial (HPLC vials). 10 μl of a 100 ng/ml solution of doxorubicin (DOX) in methanol (1 ng) was added to the vial as internal reference. The samples were evaporated under vacuum at 37 °C.

### UV–visible and MS/MS analysis

Analysis was performed by MS/MS for DNR (with 1 ng of DOX as internal reference) and UV/vis for DEX (concentration too high for MS/MS detection) at 240 nm wavelength during the first 5 weeks. After that, the amount of DNR and DEX released were both measured by MS/MS analysis.

An Agilent 1260 liquid chromatograph (LC) system coupled with a Thermo LCQ Deca mass spectrometer (MS) was used to perform the LC-MS/MS analysis using positive ion mode electrospray ionization (ESI) as the ion source with source voltage of 5 kV, sheath gas flow rate of 80 units, auxiliary gas flow rate of 20 units, and capillary temperature of 250 °C. A Phenomenex EVO C-18 column (2.1 mm ID, 5.0 mm length, 2.6 um particle size) was employed for LC separation using 2.5% methanol in water with 0.1% formic acid as the mobile phase A and pure methanol with 0.1% formic acid as the mobile phase B. The LC flow rate was set at 0.30 ml/min. The LC gradient increased from 30% mobile phase B to 95% mobile phase B in 10 min, held at 95% B for 2 min, returned to 30% B in 1 min, and then held at 30% B for 7 min. The UV detection wavelength was set at 240 nm for DEX, and 254 nm was selected for DOX. For MS/MS analysis, the data was recorded as follows: DNR: *m*/*z* 528 then SRM at *m*/*z* 381; DEX: *m*/*z* 393 then SRM at *m*/*z* 373; DOX: *m*/*z* 544 then SRM at *m*/*z* 397. A LC-ESI-MS/MS run of pure Hank’s balanced salt solution was used as a blank control.

### *In vivo* ocular safety of the dual-drug loaded pSiO_2_-DNR-DEX system

This pSiO_2_-DNR-DEX dual-drug delivery system is designed for intravitreal use. In addition to long-term release, ocular safety is the first criteria to meet. Seven pigmented New Zealand rabbits were used for this study. Three males and four females with an average age of 4.6 ± 2.4 months and an average weight of 3.52 ± 0.82 kg. All animal handling was performed according to the ARVO Statement for the Use of Animals in Ophthalmic and Vision Research, and the studies were approved by the Institutional Animal Care and Use Committee of the University of California, San Diego. One eye of each rabbit was injected with pSiO2-DNR-DEX and the contralateral eyes received equivalent diluent injections as control. The injection volume was 100 µl and the targeted dose of the delivery system was 2 mg for short-term (3 weeks) evaluation and 4 mg for long-term evaluation (3 months). A 25-gauge needle attached to a 1 ml syringe was used to inject the particles into the vitreous cavity from the pars plana (1.5–2 mm from the limbus) of the rabbit eye as reported previously by us (Chhablani et al., 2013). After seeing the injected pSi particles under direct view of a surgical microscope, the needle was kept in place for 50 s to allow for eye fluid equilibration and then the entry hole was suppressed by a cotton applicator for 1 min to avoid leaking immediately after the needle was removed. After intravitreal injection, the eyes and pSiO_2_-DNR-DEX system were monitored by ophthalmic examination including slit-lamp biomicroscopy, tonometry, and indirect ophthalmoscopy at the first week then every two weeks. Retinal function was evaluated by electroretinography (ERG) at 3 weeks and 3 months. Histology was performed for light microscopy after sacrifice.

### Statistical analysis

Intraocular pressure (IOP) and ERG parameters from the *in vivo* study belong to repeated measuring data category. A mixed model regression analysis was performed to identify the significant difference between the study eyes and the control eyes. The statistical analyses were performed by JMP software (JMP, Version 13, SAS Institute Inc., Cary, NC, 1989–2007).

## Results

### Characterization of pSiO_2_ microparticles

The dimensions of the obtained pSiO_2_ microparticles (20 µm thickness) were 40.03 ± 11.63 µm by 39.06 ± 11.50 µm as measured from light microscope images. Fifty percent of the particles fall between 30 and 45 µm. The SEM plan-view image displayed a high porosity with homogeneous pore sizes (Supplemental Figure S1).

The adsorption-desorption isotherm displays a type IV curve (Supplemental Figure S2), in agreement with the mesoporous nature of the material. The particles have a specific surface area of 193.65 ± 0.79 m^2^/g calculated with the BET algorithm, and an average pore size of 26 nm and a total pore volume of 1.05 cm^3^/g calculated with the BJH algorithm (Supplemental Figure S2).

#### Surface modifications and drug loading

Surface functionalization and subsequent covalent drug loading were characterized by FTIR spectroscopy (Supplemental Figure S3) and quantified by TGA. Oxidized particles displayed a very intense peak at 1070 cm^−1^ corresponding to the silica matrix (Si–O–Si stretch). The appearance of three peaks at 2970, 2920, and 2880 cm^−1^ assesses the effectiveness of APTES grafting (C–H2 stretch). The reaction of succinic anhydride with amines is measured by the formation of amide bonds and the appearance of the peaks at 1720, 1630, and 1560 cm^−1^ (C = O stretch) corresponding to the carbonyl groups. DNR loading leads to a slight shift of the carbonyl peaks (1740 and 1640 cm^−1^) and the FTIR spectrum displays an intense peak at 1650 cm^−1^ after DEX loading (Supplemental Figure S3).

Surface modification and drug loading were quantified by TGA and the efficiencies were determined as 4.75% loading efficiency if only DNR is loaded and 6.74% loading efficiency for dual-drug loading (Table 1). In contrast to loading DNR first, loading Dex prior to DNR yielded a loading efficiency of 5.91% for Dex; however, DNR failed to load and no DNR could be detected by TGA.

**Table 1.** Drug loading parameters.

Step	Grafting efficiency (mg/g of particles, % in mass)	Loading efficiency (mg/g of particles, % in mass)
–NH_2_	28.3, 2.83%	
–NH–COOH	42.7, 4.72%	
DNR loading		47.5, 4.75%
DEX loading		59.1, 5.91%
Dual drug loading		67.4, 6.74%

DNR: daunorubicin; DEX: dexamethasone.

### *In vitro* drug release

**Figure 2.**
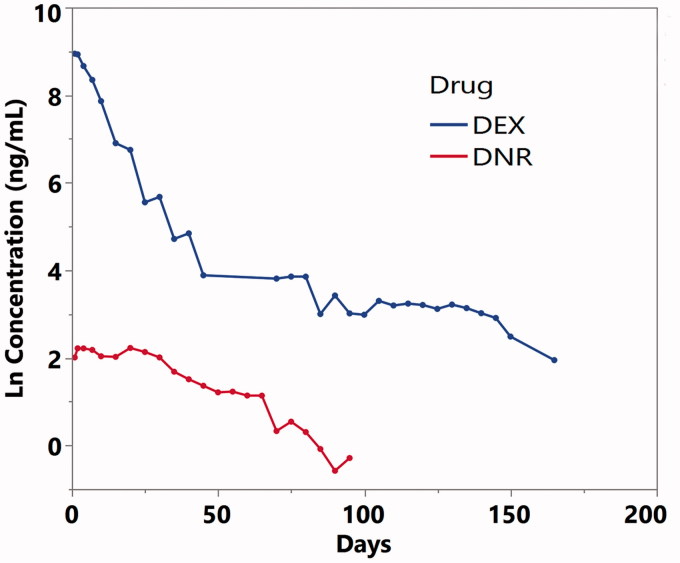
Dex and DNR concentration-time curves from in vitro release. The Y axis is in natural log scale. Dex: dexamethasone; DNR: daunorubicin.

*In vitro* release demonstrated a sustained slow release mode for both payloads, lasting for at least 3 months. The release was conducted in 1.5 ml of PBS that was replaced daily, mimicking rabbit vitreous volume and the turnover rate of vitreous fluid at 1 µl per min (Hou et al., 2014). DNR showed a 23-day steady state before an apparent elimination phase for the remaining 70 days. In contrast, DEX release demonstrated a fast release phase of 45 days followed by a slow tapering release out to 165 days (Figure 2). The concentration-time data was analyzed using WinNonlin 6 (Phoenix 64, Build 8.0.0.3176, Certara USA, Inc., 100 Overlook Center, Suite 101, Princeton, NJ 08540 USA) The key pharmacokinetic parameters from a noncompartmental analysis are summarized in Table 2.

**Table 2.** *In Vitro* release kinetics and predicted parameters.

Drugs	*R*^2^	*C*_max_ (ng/ml)	*T*_max_ (days)	Terminal Half-life(days)	AUC_last_ (ng-day*day/ml)	AUCinf_pred (ng-day*day/ml)
DEX	0.97	7661.4	1	16.6	71742.2	71910.8
DNR	0.94	9.2	20	19.2	449.3	448

AUC: area under curve; *C*_max_: maximum concentration; DEX: dexamethasone; DNR: daunorubicin; last: up to the last observed data; inf_pred: infinity predicted; *T*_max_: the time at which the concentration was maximum.

### Ocular safety of the pSiO2-DNR-DEX system

**Figure 3.**
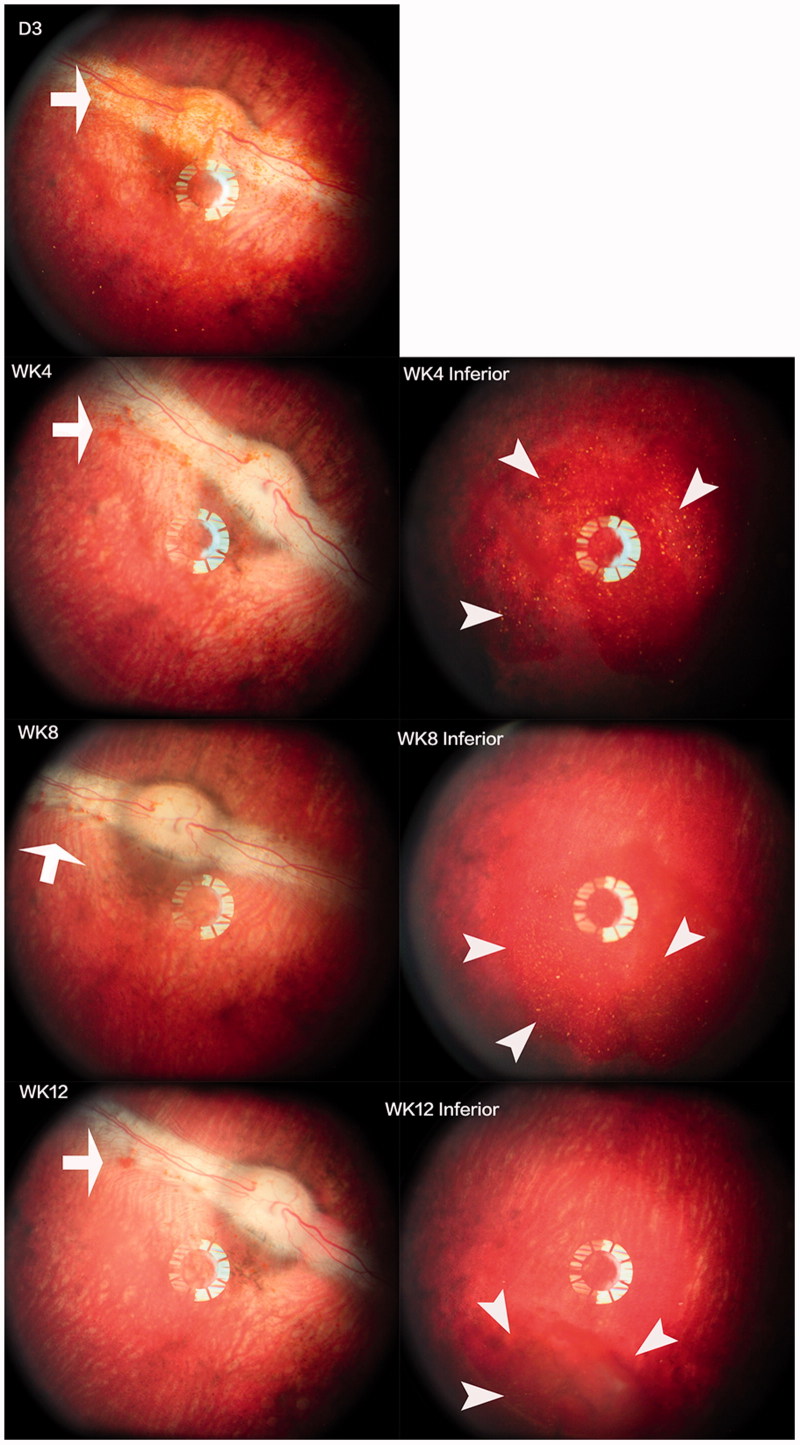
Images of the fundus and injected particles. The left column shows the optic nerve and visual streak. The right column displays the inferior view of the fundus. The injected particles appeared reddish due to the color of DNR (arrows) and aggregated into the inferior vitreous cavity (arrow heads) within a few days after the injection. Fundus photographs at all-time points showed clear vitreous and normal retina.

**Figure 4.**
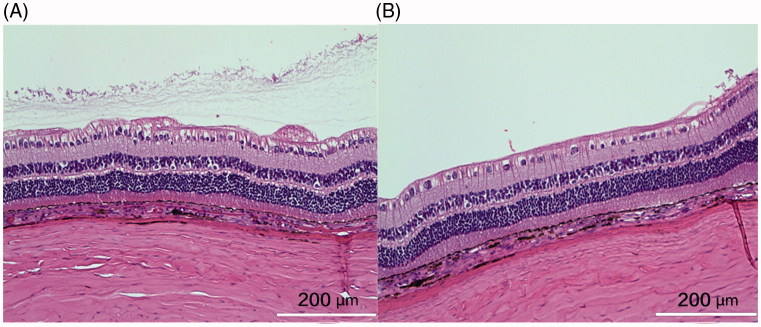
The left panel was from the control eye and the right panel from the study eye. Both sections were from similar location in relation to the visual streak. The structure of the retina appeared normal and comparable though the section from the control eye may be closer to medullary ray, showing more radiating myelinated nerve fibers on the inner surface of the retina. The cortical vitreous collagen can be seen in the vitreous near the retina of the control eye. The white bar =200 µm.

The mean injected pSiO2-DNR-DEX was 2.02 ± 0.27 mg/eye for the short-term study and 4.0 ± 0.4 mg/eye for the long-term study. No clinical toxicity was noted during the course of study. The injected particles dispersed in the vitreous, then settled down into the inferior vitreous cavity (Figure 3). There was no difference in IOP between the injected eyes and the control eyes (*p* = .9), though IOP of both eyes increased over the study course (*p* < .0001, Supplemental Figure S4). No significant difference in ERG b-wave amplitude between the injected eyes and the contralateral eyes was found (Supplemental Figure S5). The regression analysis, including ERG types and examination time points (3 weeks and 3 months), revealed that overall least square mean for the injected eyes was 67.12 µV versus 72.73 µV for the control eyes (*p* = .16). From the *in vitro* release profile, the most drug released during the first 3 weeks; however, the histology at week 3 did not show retinal toxicity (Figure 4), which confirmed the clinical findings.

## Discussion

This study demonstrated a pSiO_2_ based dual-drug delivery platform designed for intravitreal use and long-term sustained release. Distinctive from other dual-drug loaded systems, such as hydrogels (Castro et al., 2012) or dendrimers (Tekade et al., 2009), the current pSiO_2_ based system provided about 100 days of slow releasing DNR and over 165 days of slow releasing of DEX. This is the longest dual-drug release reported in the literature so far. In contrast to hydrogels or dendrimers, which utilized infiltration drug loading, the current pSiO_2_-DNR-DEX system used a covalent conjugation mechanism, which facilitates long-term release. The retinal diseases we are targeting are of chronic and relapsing nature, which require many months of sustained drug levels in the retina and choroid. Infiltration loading often fails to provide the needed therapeutic duration even when using pSi (Chhablani et al., 2013). Poly(lactic-*co*-glycolic acid) (PLGA) was used to fabricate composite mats of PLGA and mesoporous silica nanoparticles in which one model drug was loaded into the nanoparticles and the other was loaded into the PLGA (Song et al., 2012). Such a compound dual-drug system released its drugs for only 2 weeks (Song et al., 2012). pSi nanoparticles were also used to load the anti-cancer drug methotrexate using covalent conjugation followed by infiltration loading of another therapeutic, sorafenib (Wang et al., 2015). The nanoparticles were designed to be internalized into cancer cells and release the drugs. In *in vitro* release, methotrexate was depleted within the first four days (Wang et al., 2015).

The current pSiO_2_-DNR-DEX dual-drug delivery system utilizes a covalent loading strategy for both payloads. This integrated two-drugs on one-particle system has an advantage over co-injecting a mixture of two single-drug loaded pSiO_2_ particles. The current dual-drug delivery system delivers the hydrophilic DNR and the hydrophobic DEX simultaneously. It is well known that hydrophobic drug loaded particles have very poor syringeability that can cause inaccurate dosing. Due to the sharply different syringeabilities of two single-drug loaded formulations, the targeted optimal ratio of two therapeutics will be difficult to realize. In contrast, a single dual-drug loaded delivery system offers the ability to ensure a consistent ratio of the two therapeutics while injecting less inert material into the eye.

The two current model drugs are FDA-approved therapeutics and are being used clinically as free drugs (Wiedemann et al., 1998) or in an intravitreal implant (Schwartz et al., 2016). In this study, drug quantitation from the dissolution medium was performed by mass spectrometry which confirmed that the eluted drugs retained their original structures. Though their efficacy on ocular angiogenesis and inflammation is well known, little is known about the optimal drug concentration of these two therapeutics in a synergetic setting or within a prolonged period of presence in the eye. DNR has been reported to be toxic to the retinal pigment epithelium *in vitro* with an EC50 of 10 µM after 24 h of exposure (Hueber et al., 2003). However, if the exposure is increased to 7 days, 50 ng/ml can induce significant cytotoxicity (Garweg et al., 2006). The EC50 for doxorubicin, a drug chemically and pharmacologically similar to DNR, was reported to be 10 ng/ml (Blumenkranz et al., 1984). Considering the possible synergistic effect and sustained release, we tried to deliver a low concentration of DNR to avoid retinal toxicity. *In vitro* study has shown the EC90 is about 40 ng/ml for DEX (Jaffuel et al., 2001); however, DEX has a much better ocular safety profile and intravitreal toxicity was reported to be 440 µg (Kwak & Damico, 1992). Therefore, the current dual-drug pSiO_2_ delivery system seems to release safe and therapeutically relevant concentrations of DNR and DEX. Though their efficacy on retinal diseases has been clinically attested to manage vitreoretinal proliferation and inflammation (Wiedemann et al., 1998; Calvo et al., 2015), the current dual-drug delivery pSiO_2_ platform needs to be tested in animal models to confirm its synergistic efficacy. The superiority of synergistic therapy has been demonstrated in cancer therapy (Feng et al., 2017; Xu et al., 2017b). In fact, it may be possible that due to synergistic action, lower drug concentrations are required than would be when administered separately. With the drug loading technology developed in this investigation, it is possible to tune each drug-loading efficiency by controlling the loading time ratio of the two drugs. In this study, the loading time was 2 h for DNR and 7 days for DEX. It is also interesting to note that switching the loading order (loading of DEX prior to DNR) led to significantly less DNR in the dual-drug delivery system. A logical explanation to this may be that DEX is highly hydrophobic and this property is transferred to the pSiO_2_ particles after loading. The subsequent loading of DNR (hydrophilic) occurs in water using water-soluble coupling agents (EDC and sulfo-NHS). Thus, the acquired hydrophobicity of the DEX loaded particles acts as a shield, repelling water and forbidding access to EDC and sulfo-NHS, which are mandatory to activate COOH functions before DNR coupling. Moreover, it may also prevent DNR from reacting with the functionalized surface.

In summary, we demonstrated a dual-drug delivery system based on pSi. This drug delivery system showed good ocular biocompatibility and therapeutically relevant drug release rates of two drugs. This technology also opens the door for the creation of a dual-drug loaded system utilizing nonsteroidal anti-inflammatory drugs such as ketorolac instead of DEX in order to eliminate steroid-related ocular side effects. Further *in vivo* efficacy studies in animal eye disease models are warranted to optimize and characterize this dual-drug delivery platform.

## Supplementary Material

Supplemental Figure S5

Supplemental Figure S4

Supplemental Figure S3

Supplemental Figure S2

Supplemental Figure S1
