# Supplementary figures and images for: Porous silicon based intravitreal platform for dual-drug loading and controlled release towards synergistic therapy
Source: Drug Deliv. 2018 Jul 11;25(1):1537–45. doi: 10.1080/10717544.2018.1486474 (PMC6058705; doi:10.1080/10717544.2018.1486474)

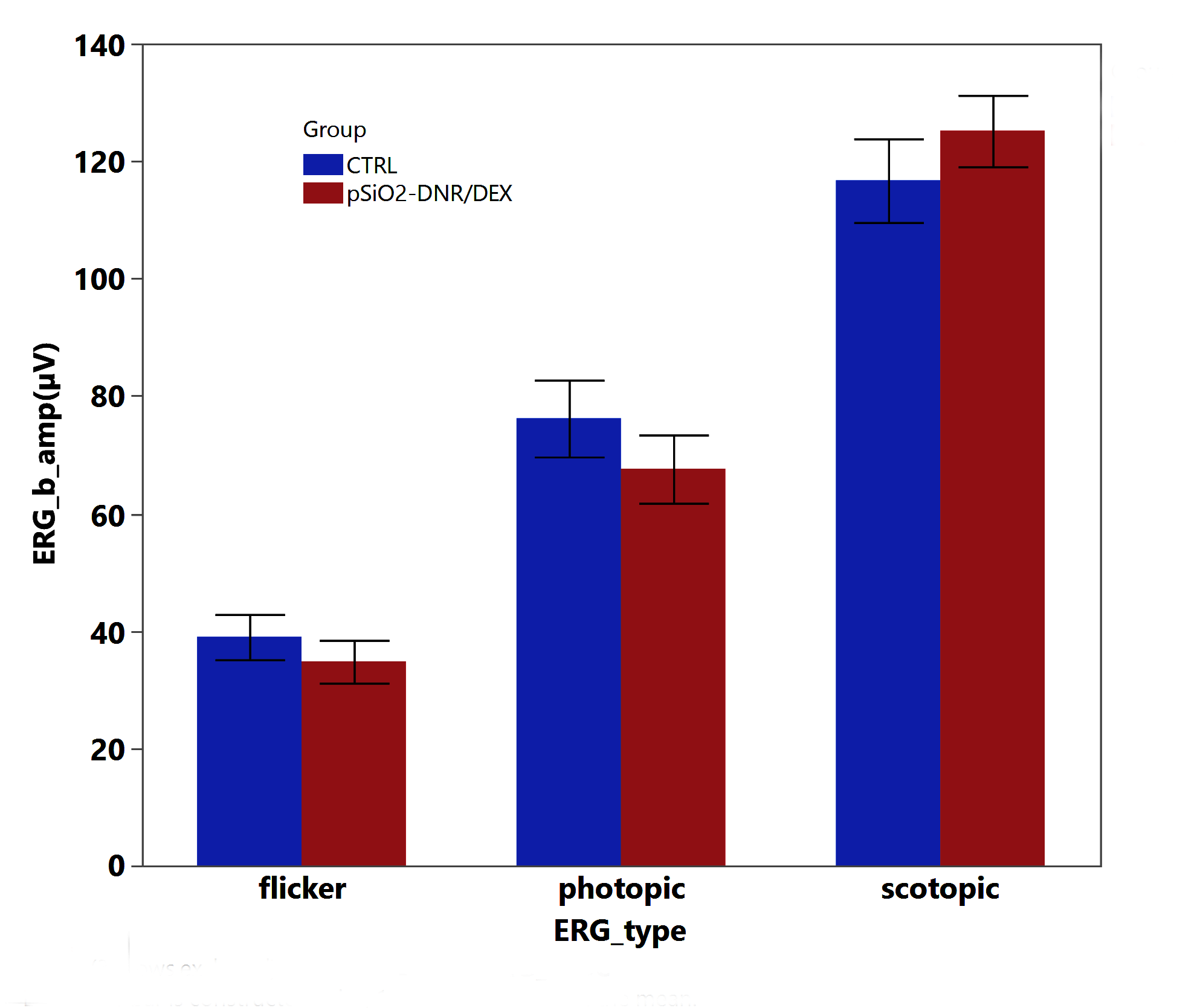

Supplement: Supplemental Figure S5 [file IDRD_A_1486474_SM6932.tif]

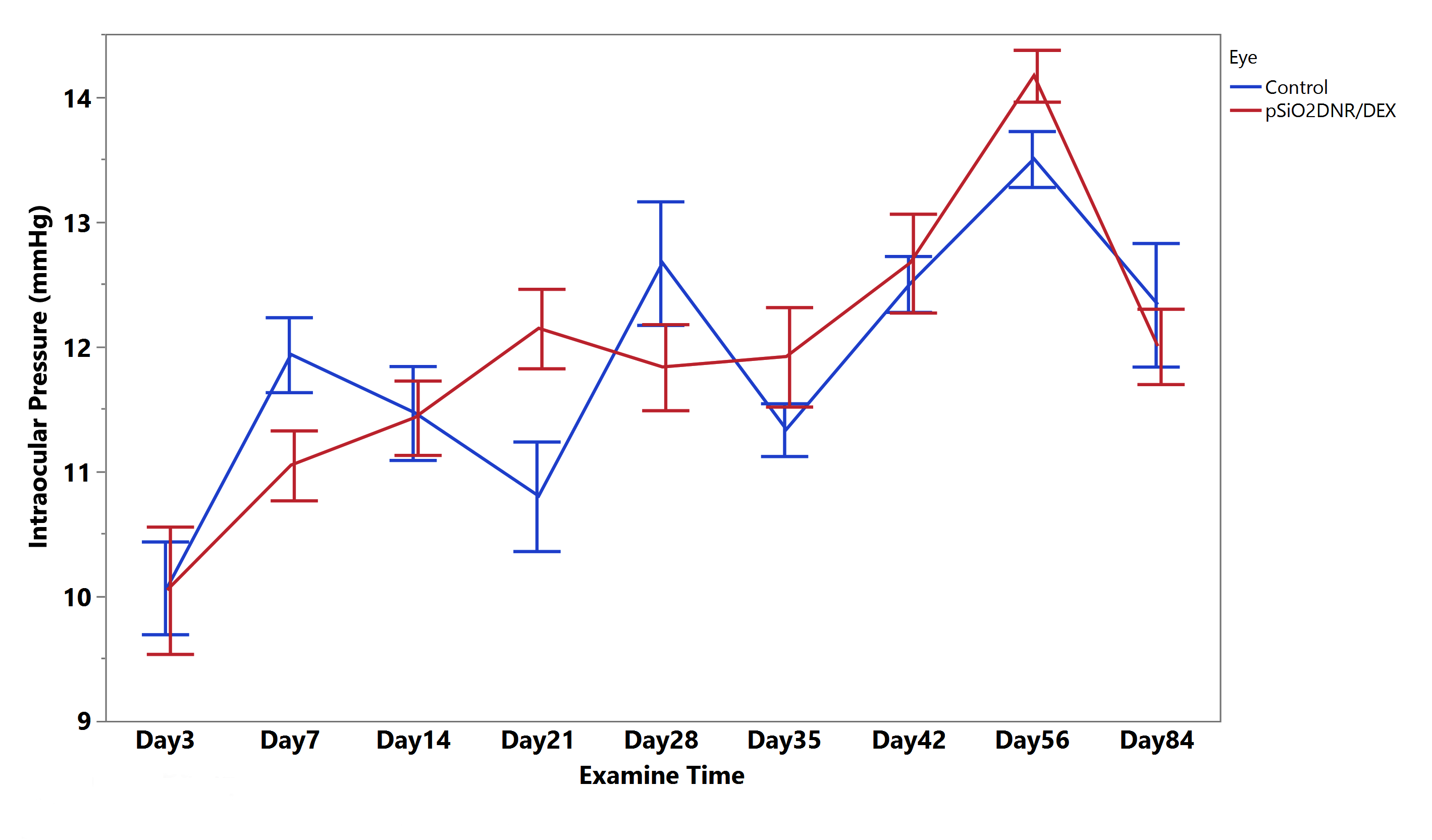

Supplement: Supplemental Figure S4 [file IDRD_A_1486474_SM6931.tif]

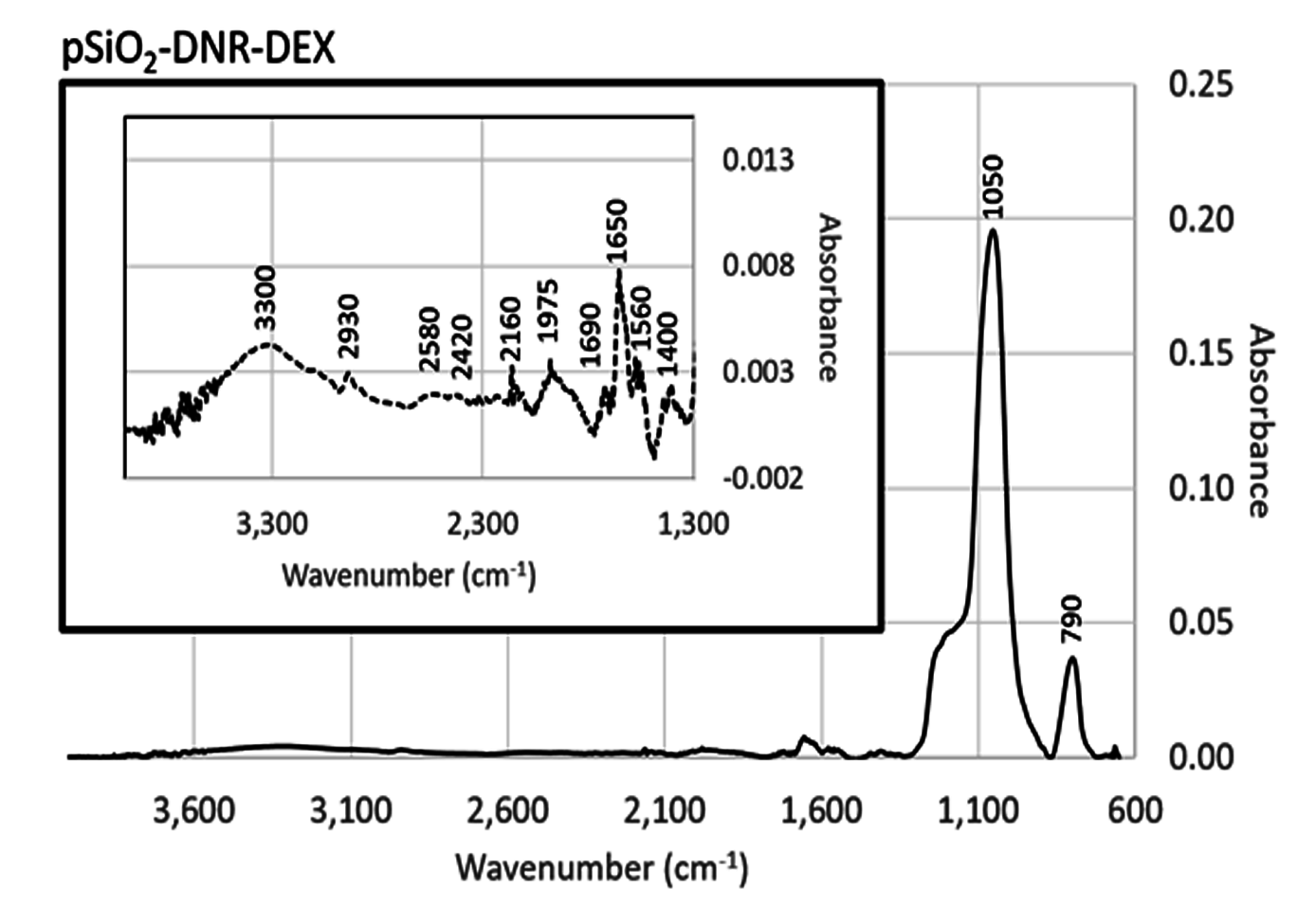

Supplement: Supplemental Figure S3 [file IDRD_A_1486474_SM6930.tif]

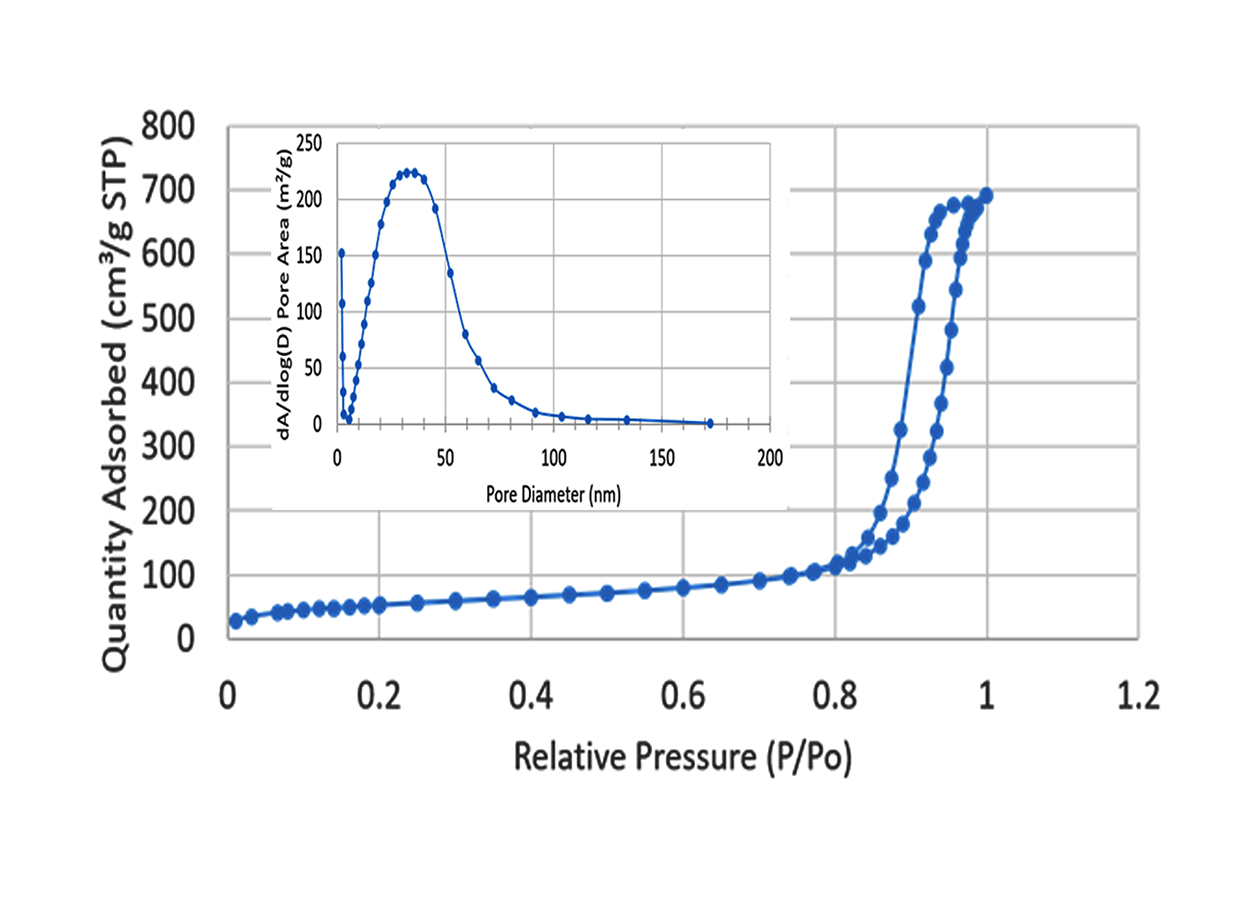

Supplement: Supplemental Figure S2 [file IDRD_A_1486474_SM6929.tif]

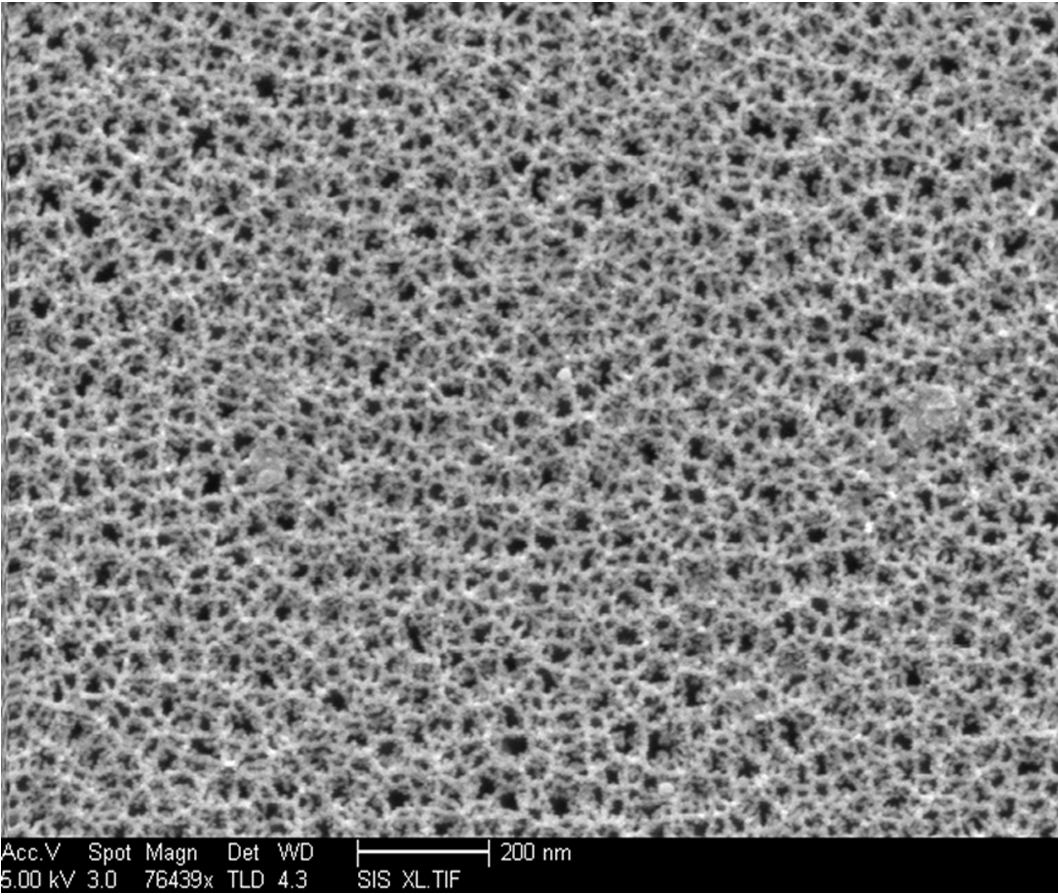

Supplement: Supplemental Figure S1 [file IDRD_A_1486474_SM6928.tif]
